# Bisphenol A Effects on Mammalian Oogenesis and Epigenetic Integrity of Oocytes: A Case Study Exploring Risks of Endocrine Disrupting Chemicals

**DOI:** 10.1155/2015/698795

**Published:** 2015-08-03

**Authors:** Ursula Eichenlaub-Ritter, Francesca Pacchierotti

**Affiliations:** ^1^Faculty of Biology, Gene Technology/Microbiology, University of Bielefeld, 33601 Bielefeld, Germany; ^2^Laboratory of Toxicology, Unit of Radiation Biology and Human Health, ENEA CR Casaccia, 00123 Santa Maria di Galeria, Rome, Italy

## Abstract

Bisphenol A (BPA), originally developed as a synthetic oestrogen, is nowadays extensively used in the production of polymeric plastics. Under harsh conditions, these plastics may release BPA, which then can leach into the environment. Detectable concentrations of BPA have been measured in most analysed samples of human serum, plasma, or urine, as well as in follicular fluid, foetal serum, and amniotic fluid. Here we summarize the evidence about adverse BPA effects on the genetic and epigenetic integrity of mammalian oocytes. We conclude that increasing evidence supports the notion that low BPA concentrations adversely affect the epigenome of mammalian female germ cells, with functional consequences on gene expression, chromosome dynamics in meiosis, and oocyte development. Specific time windows, during which profound chromatin remodelling occurs and maternal imprints are established or protected, appear particularly vulnerable to epigenetic deregulation by BPA. Transgenerational effects have been also observed in the offspring of BPA-treated rodents, although the epigenetic mechanisms of inheritance still need to be clarified. The relevance of these findings for human health protection still needs to be fully assessed, but they warrant further investigation in both experimental models and humans.

## 1. Introduction

According to the “Developmental Origin of Health and Disease” hypothesis (DOHaD) that was proposed over a decade ago [1, 2], lifestyle, nutrition, and exposures during pregnancy can influence the health of the offspring from birth to much later in life. The DOHaD hypothesis proposes that famine, nutritional deficits, or diabetes in the mother can predispose the offspring to diseases or reduce its fertility, especially if exposures occur during critical periods of embryogenesis or foetal development [3–5]. Prenatal exposure of the developing germline may entail the additional risk to transmit the induced damage to the following generation.

Environmental exposures that affect metabolism or hormonal homeostasis do not necessarily induce DNA mutations but may influence gene expression by disturbances in epigenetic regulation. Recently, it has been shown in rodents that* in utero* undernourishment alters the germline DNA methylome of *F*1 adult males in a locus-specific manner. Although altered DNA methylation did not persist in *F*2 tissues, dysregulated expression of genes neighbouring affected loci was observed, suggesting the possibility of intergenerational transmission of environmentally induced disease not mediated by Mendelian inheritance [6].

According to the US Environmental Protection Agency, endocrine disrupting chemicals (EDCs) are defined as “exogenous agent(s) that interfere(s) in synthesis, secretion, transport, metabolism, binding action, or elimination of natural blood-borne hormones that are present in the body and are responsible for homeostasis, reproduction, and developmental processes” [7]. As such they may transiently alter gene expression patterns in exposed cells, organs, and individuals by interfering in hormonal homeostasis, for example, by acting as agonist or antagonist in hormone receptor-mediated signalling. Moreover, it has recently been shown that exposure to EDCs may induce transgenerational phenotype alterations, possibly caused by differential DNA methylation in gene promoter regions, termed epimutations [8–12].

## 2. Bisphenol A

**Figure 1:**
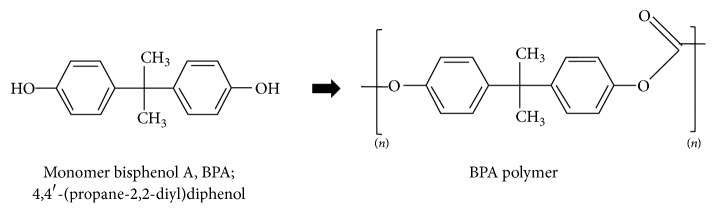
BPA monomer and polymer.

Monomer bisphenol A (4,4′-(propane-2,2-diyl)diphenol) (BPA) (Figure 1) was developed in 1891 as a synthetic oestrogen (xenoestrogen). BPA indeed binds to oestrogen receptors* in vivo* and* in vitro* [13], but, due to its low oestrogenic activity (≈10^3^–10^5^ less than the natural steroid, oestradiol), it was replaced by diethylstylbestrol (DES), which had much stronger oestrogenic properties. DES is sadly known for the teratogenic and carcinogenic effects observed in the genital organs of daughters of women using DES in pregnancy to prevent spontaneous abortion [14]. Recently, an epigenetic influence of DES on the regulation of histone [15] and DNA [16] methyltransferases has been shown that could play a role in the induction of its reproductive effects. The case of DES could be considered an alarming sentinel of the importance of epigenetic mechanisms in EDCs adverse effects.

Even though BPA was not marketed as a hormonal active substance, in the last decades, it found application as plasticizer in the production of polymeric plastics, mainly polycarbonate (71%) and epoxy resins (29%) [17]. For a long time, polymeric BPA was considered harmless, as it does not interact with steroid receptors (Figure 1). Over the last 50 years, the use of BPA-containing polymers in common items such as plastic bottles, toys, lining of aluminium cans and pipes, dental sealants, and thermal receipt paper led to increasing BPA production which reached about 5 million tons in 2010 [17]. Unfortunately, polycarbonate plastics damaged by heat, UV, harsh alkaline treatment, or after vigorous washing were shown to release monomeric BPA. By now, it is estimated that the worldwide release of BPA into the environment is exceeding one million pounds/year [18].

## 3. Environmental and Human BPA Contamination

In the USA, average BPA groundwater concentrations range between 0.0041 and 1.9 mg/m^3^, and up to 20 mg/m^3^ BPA were measured in some areas of Great Britain [17]. BPA can be efficiently biodegraded in water and soil by microorganisms and by photolysis in water at wavelengths above 290 nm [17, 19, 20]. However, in spite of environmental biodegradation, 0.1–790 *μ*g/kg BPA were detected in fresh weight (f.w.) food and up to 0.86 mg/m^3^ in drinking water or commercial drinks [17]; biomonitoring studies detected BPA in human serum, plasma, or urine of over 90% US and Canadian citizens [21]. Daily dietary BPA intakes of about 0.02–0.08 *μ*g/kg/day and 0.22–0.33 *μ*g/kg/day have been estimated for adults and infants, respectively (for references see [17]). BPA is rapidly metabolised to bisphenol A-glucuronide in liver, but unconjugated BPA has been detected in serum and blood of the general population at concentrations of 4.4 mg/L and 2.5 mg/L, respectively, and over 50 mg/L BPA were measured in workers [18, 22]. BPA has been also detected in follicular fluid, foetal serum, and amniotic fluid (average 1-2 ng/mL) [23] and in umbilical cord serum of human mid gestation embryos [24].

## 4. BPA Effects on Mammalian Oogenesis

Although potential adverse effects of low BPA concentrations on reproduction are still a matter of debate, most studies suggest that BPA is an ovarian toxicant and reduces oocyte quality in animal models and in humans [23, 25]. Potential mechanisms of BPA action on hormonal homeostasis include binding to nonclassic membrane oestrogen receptors (mERs), binding to glucuronide receptor, activation of nuclear oestrogen-related receptor gamma (ERR*γ*), suppression of thyroid hormone receptor transcription, decrease of cholesterol transport through the mitochondrial membrane, increase of fatty acid oxidation, stimulation of prolactin release, and impairment of aromatase expression (reviewed in [18, 25–27]).

**Figure 2:**
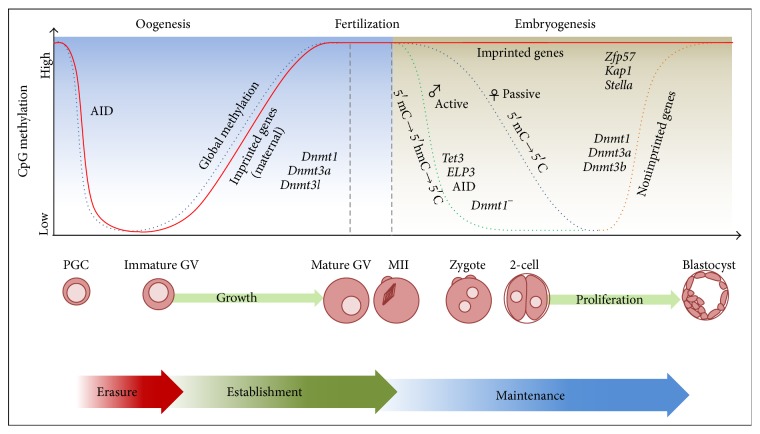
Critical stages for epigenetic reprogramming of chromatin in mammalian female germ cells, including periods of imprint erasure during formation of primordial germ cells, maternal imprint establishment during oocyte growth, and imprint maintenance after fertilization of the egg and development to the blastocyst. Statuses of maternal imprints are indicated by red lines, of global methylation in maternal chromatin in dotted blue lines, of global paternal methylation in green dotted lines, and of development and tissue-specific methylation in orange dotted lines. Some enzymes that participate in demethylation (activation induced cytidine deaminase), DNA methylation (Dnmts), or maintenance of methylation (zinc finger protein 57, Zfp57; tripartite motive containing, Kap1/Trim28; developmental pluripotency associated 3, Stella/Dppa3) are indicated next to the respective lines showing changes in DNA methylation, adapted from [84].

Mammalian oocytes are amongst the most long-lived cells in the body. Primordial germ cells start differentiating already in the early postimplantation embryo, after migration to the genital ridges (Figure 2) [28]. Nests of primary oocytes, entering meiosis I, are formed in the human ovary by the 3rd month of pregnancy. Pairing and recombination between homologous chromosomes take place in the foetal ovary before birth. Around the 7th month of pregnancy, oocytes finally develop to the late dictyotene stage, when they become meiotically arrested. By that time, the synaptonemal complexes have disappeared and the homologous chromosomes remain attached by one or more meiotic exchanges and chiasmata until much later in oogenesis when oocytes resume meiosis in the sexually adult female; in the meantime the sister chromatids of each chromosome remain tightly linked by cohesion complexes (reviewed in [29]).

Exposure of mice, from midgestation until birth, to daily doses of 400 ng BPA resulted in synaptic abnormalities and increased rates of recombination between homologous chromosomes in the oocytes. Interestingly, these effects resembled those observed in mice homozygous for a targeted disruption of the gene encoding for oestrogen receptor *β* [30]. Increased recombination was also observed in oocytes of rhesus monkeys prenatally exposed to BPA [31] and in human oocytes treated* in vitro* with BPA [32]. Moreover, the expression of genes involved in recombination and DNA repair was altered in the BPA-exposed human foetal oocytes [33]. It is known that alterations in the number and localization of chiasmata can adversely affect chromosome segregation and predispose the oocytes to aneuploidy (reviewed in [29]). The studies on altered recombination in BPA-exposed foetal oocytes therefore suggest that the female offspring of BPA-exposed mothers might be at risk for meiotic chromosome nondisjunction.

In addition to effects on chromosome synapsis in the oocytes, BPA was shown to induce also alterations of follicle maturation. Normally, at birth, the oocyte nests break down and primordial follicles are formed by recruitment of a single layer of flattened granulosa cells around the dictyate-arrested oocytes. Primordial follicles will develop to the secondary, tertiary, and finally large antral stage only from puberty onwards, when folliculogenesis proceeds under the influence of gonadotropic hormones (follicle stimulating hormone, FSH, and luteinizing hormone, LH). Mature follicles contain multilayered outer mural granulosa cells and layers of cumulus granulosa cells surrounding a fully grown, meiotically and developmentally competent oocyte, within a large fluid-filled space, the antrum. The follicle is surrounded by a basal membrane and by layers of luteal cells that are involved in steroidogenesis. Growth of the oocyte and full development of the follicle is a lengthy process that takes about 120 days in humans.

In rhesus monkeys, chronic exposure to BPA during pregnancy, leading to serum concentrations of 2.2–3.3 ng/mL, caused in the offspring a significant increase in the frequency of abnormal follicles containing multiple oocytes [31]. Disturbances in nest breakdown and primordial follicle formation were also noted in mice upon* in utero* exposure [34].* In vitro* experiments supported the notion that BPA impairs follicular development. Germ cell nest breakdown and primordial follicle assembly were significantly reduced when newborn mouse ovaries were exposed to 10 or 100 *μ*M BPA in culture medium [35]. Similarly, 100 *μ*g/mL BPA (440 *μ*M) inhibited follicle growth and induced atresia in a mouse follicle* in vitro* model [36], by mechanisms that were independent of the genomic oestrogenic pathway [37]. Continuous 12-day exposure of mouse follicles in culture, from the early preantral up to the large antral stage, to 30 *μ*M BPA led to reduced granulosa cell proliferation and arrest of some oocytes at meiosis I [38], whereas lower BPA concentrations did not significantly affect follicular development and hormone release. Finally,* in vitro* treatment of human oocytes with low BPA concentrations was shown to increase the frequency of oocyte degeneration [32].

Since the entire pool of primary oocytes that can ever be ovulated is already formed in the foetal ovary, disturbances in oocyte meiosis and follicle formation, recruitment and survival, induced by any mechanism, can contribute to premature ovarian depletion, a clinically recognized condition in women (termed “premature ovarian insufficiency,” POI). In addition, the fidelity of chromosome segregation at first and second meiosis might be compromised when the oocyte pool becomes prematurely depleted. Whether BPA exposure may influence follicle pool size in humans is still controversial. High urinary BPA levels were associated with reduced antral follicle counts in a cohort of 209 women undergoing infertility treatments [39], whereas no correlation was found between serum BPA levels and antral follicle counts in another study on a smaller cohort of 44 patients [40]. Nevertheless, several data suggest a negative impact of BPA on woman fertility. Urinary BPA levels were negatively correlated with numbers and quality of oocytes retrieved in stimulated cycles for assisted reproduction [41, 42]. Increased urinary [41, 42] or serum [43] BPA concentrations were also associated with decreased peak oestradiol levels. Finally, a study on 137 patients undergoing assisted reproduction suggested that high urinary BPA levels might be associated with up to 50% higher chance of implantation failures, in comparison to patients with low or no evidence of BPA exposure [44].

Once follicles have developed to the large antral, Graafian stage, release of one (in monoovulatory species, like humans) or of multiple (in multiovulatory species like rodents) oocytes from meiotic arrest occurs under the influence of gonadotropins and the LH surge, downstream from signalling by complex and redundant pathways. Resumption of meiosis I, normally, results in gene expression changes and molecular signalling in cumulus cells and oocyte via epidermal growth factor-like hormones and critical changes in the concentration of cyclic nucleotides. Fully grown, developmentally competent oocytes then become transcriptionally quiescent, and their chromatin is remodelled to surround the nucleolus in a characteristic fashion [45]. Following the LH surge, the maturation promoting factor/cyclin-dependent kinase 1 pathway of the oocyte becomes activated, chromatin becomes condensed, and histones are characteristically deacetylated and posttranslationally modified in specific ways [46]. A spindle is then formed in the ooplasm, and oocytes complete the first meiosis with the reductional division of homologous chromosomes, reach the metaphase II stage, when they arrest again, and are finally ovulated, surrounded by the expanded cumulus complex.

In 2003, it was reported that low, chronic BPA doses might induce aneuploidy in oocytes exposed prior to resumption of meiosis [47]. Oral treatment of mice with 20, 40, or 100 ng/g b.w. BPA, for 6–8 days prior to isolation and* in vitro* maturation of oocytes, induced meiotic arrest, spindle abnormalities and misalignment of metaphase II chromosomes. These experimental results supported the hypothesis that a sudden, unexpected increase of aneuploid oocytes that had previously occurred in the mouse colony had been caused by accidental release of BPA from damaged plastic bottles and cages. An independent study, conducted under similar exposure conditions to verify these findings, showed BPA induction of subtle spindle abnormalities in metaphase II oocytes, but not aneuploidy [48]. Similarly, no evidence of aneuploidy induction was obtained in metaphase II oocytes collected from mice treated with a single BPA dose, with 7 daily administrations or exposed for 7 weeks to BPA in drinking water [49]. Differences in the animal diet were suggested to explain these inconsistencies when it was shown that the phytoestrogen content in animal feed could influence the rate of spindle aberrations induced in metaphase II oocytes by 7 daily low dose administrations of BPA [50]. Other studies showed an influence of the diet on BPA-induced changes in DNA methylation [51], supporting an interaction between BPA biological activity and dietary factors.

*In vitro* experiments in mouse oocytes showed that high concentrations of BPA induced spindle aberrations, chromosome congression abnormalities, and meiotic arrest, but not aneuploidy [48, 52], suggesting that an efficient spindle assembly checkpoint was able to prevent chromosome segregation errors in healthy young oocytes. An inverse relationship between BPA concentration and percentage of oocytes that progressed to metaphase II and a dose-dependent increase in aberrant spindles and unaligned chromosomes at metaphase II were also reported for human oocytes exposed* in vitro* to 20, 200 ng/mL, or 20 *μ*g/mL BPA (88, 880 nM, 88 *μ*M) [53].

Fertilization triggers release of metaphase II oocytes from second meiotic arrest. This entails second polar body extrusion and completion of oocyte second meiosis during which sister chromatids separate from each other (reviewed in [29]). There is a paucity of data about BPA effects on the second meiotic division in oocytes. Chronic exposures of mice to 0.5 mg/L BPA in drinking water resulted in the premature separation of sister chromatids in their metaphase II oocytes, which, however, had no consequence upon the fidelity of chromosome segregation during the second meiotic division, as demonstrated by the normal chromosome constitution of zygotes under the same exposure condition [49].

## 5. BPA Epigenetic Effects on Female Germ Cells and Their Consequences

Oogenesis, from primordial germ cell differentiation to fertilization, and preimplantation embryonic development entail profound epigenetic changes (Figure 2). After global DNA demethylation in primordial germ cells, female specific genomic imprinting is set during oocyte development in a site-specific sequential fashion in imprinting control regions (ICRs). The whole process is completed prior to resumption of meiosis [54–56]. The zygote and the early preimplantation embryo are further subjected to extensive chromatin remodelling and DNA methylation changes: active hydroxymethylation and global DNA demethylation in the male chromatin and passive global demethylation in the female chromatin (Figure 2). Most of the enzymes for these events are maternally provided by the oocyte before full zygotic gene activation. The remodelling and epigenetic changes in male and female chromatin proceed according to a highly regulated sex-specific program [57], which spares the removal of DNA methylation on genomic imprints from sperm and oocyte that are important for normal preimplantation development and are retained in tissues to regulate monoallelic gene expression from paternal or maternal alleles [58, 59]. Disturbances in genomic imprinting and in DNA methylation pattern or histone pattern and chromatin conformation can contribute to epigenetic diseases such as the Angelman, Beckwith-Wiedemann, Prader-Willi, and Russell-Silver syndromes [60–62] and predispose the offspring, or even the following generations, to cancer and other diseases related to epigenetic instability.

The first report showing BPA epigenetic effects came from studies in the agouti viable yellow (*A* ^vy^) mutant mouse model. The *A* ^vy^ allele carries an Intracisternal A Particle (IAP) retrotransposon insertion upstream of the locus. The* Agouti* gene is usually expressed during a narrow window in embryogenesis and codes for a signalling molecule that can lead to either production of black eumelanin or yellow pheomelanin from a hair-cycle-specific promoter in exon 2 [63, 64]. In the *A* ^vy^ allele, transcriptional control of the* Agouti* coding sequence is driven by promoter elements in the retrotransposon, containing 9 CpGs, whose methylation level can vary among individual isogenic mice [51, 64, 65]. Hypomethylation of the 9 CpGs leads to the binding of the agouti protein to the melanocortin 4 receptor in all tissues, ectopic gene expression, and shifting of the coat colour from wild type pseudoagouti brown, to mottled, to yellow. In addition, the overproduction of the agouti protein associated to hypomethylation causes obesity, diabetes, and tumorigenesis in adult mice through its multiple actions on gene expression.

When *a*/*a* female mice received a phytoestrogen-free diet doped with 50 mg/kg b.w. BPA in the weeks prior to mating with *A* ^vy^/*a* males and throughout the gestation and lactation time periods, coat colour shift towards yellow and obesity were observed in the *A* ^vy^/*a* heterozygous offspring. These effects were related to decreased methylation in the 9 CpG sites within the *A* ^vy^ allele. Dietary supplementation with either methyl donors (folic acid, betaine, vitamin B12, and choline) or phytoestrogen prevented hypomethylation, resulting in more offspring with brown or mottled brown coat colour and normal weight [51]. Although the altered phenotype of BPA-exposed mice could be transgenerationally transmitted, the CpG methylation pattern was not inherited in the blastocyst, suggesting that other epigenetic mechanisms, like histone-mediated chromatin alterations, might be responsible for the transgenerational effects in this model.

The insulin-like growth factor II receptor (*Igf2r*) and the paternally expressed gene 3 (*Peg3*) are first imprinted in female mice after birth when follicles and oocytes start to develop in the ovary. Chao and coworkers exposed CD-1 mice to low doses of BPA (20 or 40 *μ*g/kg b.w.) either by daily hypodermal injections from postnatal day 7 to postnatal day 14 or by intraperitoneal injections administered each fifth day between postnatal days 5 and 20 [66]. BPA not only dose-dependently inhibited methylation of* Igf2r* and* Peg3* differentially methylated regions, but also lowered the transcription of DNA methyltransferase* Dmnt1*,* Dmnt3a*,* Dmnt3b* and* Dnmt3l* genes in the oocytes. Since the oestrogen receptor (ER) may recruit coactivator complexes with histone acetyltransferase or methyltransferase activities to activate downstream target genes [67], Chao and coworkers examined the expression of ERs and found a significant increase in ER*α* mRNA and protein levels at the highest BPA dose. ER inhibitor ICI182780 abolished the reduction in Dnmt gene expression in the ovary of BPA-exposed mice. These observations suggested that ER signalling mediated the epigenetic effects induced by BPA in the oocytes [66].

Further evidence for BPA effects on methylation in differentially methylated regions of imprinted genes during oogenesis and early embryogenesis came from [68]. Exposure of mice to BPA (10 *μ*g/kg or 10 mg/kg b.w.) during late stages of meiosis and oocyte growth, from 2 weeks prior to mating until day 9.5 of gestation, resulted in significant alterations in the expression of imprinted genes* Peg3*,* Snrpn*,* H19/Igf2*, and* Kcnq1* in embryonal and placental tissues and affected foetal, placental, and postnatal development. The higher BPA dose disrupted the parental specific, monoallelic expression of the* Snrpn*,* Igf2* and* Kcnq1ot1* genes in a tissue-specific manner, and resulted in the biallelic expression of the paternally expressed* Snrpn* gene in the placenta, suggesting that maternal imprinting of* Snrpn* was disturbed before fertilization or that* Snrpn* was susceptible to loss of imprinting during early embryogenesis. Expression of the normally repressed maternal allele of* Kcnq1ot1* in the placentas ranged from 12.3 to 72.3% of total expression, whereas no differences were found between control and BPA-treated mice on the* Kcnq1ot1* maternal allele expression in the embryo [68], suggesting loss of methylation control in a tissue-specific fashion. The low BPA dose did not significantly affect imprinted gene expression except for* Snrpn* and* Kcnq1ot1* loci in the placenta. To determine whether the altered expression pattern of imprinted genes was linked to abnormal DNA methylation, DNA methylation was analysed from the placentas and embryos of mice exposed to the high BPA dose during oocyte growth and early embryogenesis. A small but significant (*P* < 0.05 through ANOVA) reduction in the mean methylation level of the* Snrpn* imprinting control region was detected by pyrosequencing; furthermore, the decrease of methylation was attributed to the normally hypermethylated maternal allele by bisulfite mutagenesis sequencing, which allows assaying allele-specific methylation levels. Pyrosequencing analysis of the* H19/Igf2* imprinting control region in BPA-exposed embryos revealed also a slightly, but significantly, reduced average methylation of the 6 analysed CpG sites (*P* < 0.001). Analysis of global DNA methylation by Luminometric Methylation Assay (LUMA) in control and BPA-exposed samples found a significant difference in the placentas, but not in 9.5-day embryos, after exposure to the high dose only (*P* < 0.05). Exposure of females to the high BPA dose only from day 5.5 to day 12.5 of gestation, that is, outside of the critical windows of DNA methylation acquisition in the oocytes and epigenetic reprogramming in embryos, did not significantly affect expression of imprinted genes. As might be expected from the important role of imprinted genes in placental development, aberrant imprinting induced by BPA exposure was associated with abnormal placental phenotypes [68]. In conclusion, this study revealed that exposure to environmentally relevant doses of BPA during critical windows of oocyte development and growth and early embryogenesis can perturb expression and methylation of imprinted genes with the most significant effects observed in the placenta. It remains to be established whether loss of imprinting* per se* and/or disturbance of imprint maintenance were due to direct effects on the early embryo/placenta, or were preprogrammed in the oocyte, prior to conception.

Other studies, although not specifically focused on oocytes, support the hypothesis that BPA exposure may affect methylation of cytosines in DNA of imprinted and nonimprinted genes, outside and within coding regions. A genome-wide analysis showed that perinatal exposure to 50 *μ*g/kg or 50 mg/kg BPA in diet induced nonmonotonic dose-dependent alterations of DNA methylation patterns in liver. Altered methylation was predominantly found within CpG island shores, and, overall, several hundred novel BPA-sensitive methylation sites were identified involving pathways in metabolism and stimulus response [69].

In another study it was shown that exposure of pregnant mice throughout gestation to low doses of BPA (20 *μ*g/kg b.w.) altered the epigenome in the forebrain of the offspring, inducing hypomethylation at* NotI* locus, and deregulation of gene expression [70]. Transgenerational changes in behaviour were also noted in mice upon gestational BPA exposures [71].

An epigenetic impact of BPA was demonstrated also on male germ cells. Male offspring of rats perinatally exposed to BPA had reduced sperm counts and other changes in phenotypes not only in the first but also in the *F*3 generation [9, 10]. Induction of sperm epimutations and male-mediated transgenerational inheritance of obesity and reproductive disturbances were also shown after BPA exposure of rats [12, 72]. When female mice were exposed during gestation and lactation to low BPA doses (40 *μ*g/kg b.w.) deregulated glucose homeostasis in the *F*2 generation was observed; decreased global methylation and differential methylation of a specific CpG site in the glucokinase promoter in the *F*1 sperm suggested that the *F*2 phenotype could be caused by epigenetic alterations induced in the male paternal germline by BPA prenatal exposure [73].

Finally, exposure to BPA appears to affect DNA methylation also in humans; a study in human foetuses found an organ-specific association between changes of global DNA methylation and BPA exposure [74]; a cross-sectional study of epigenomic alterations in prepubescent girls from Egypt revealed that increasing urinary BPA levels were associated with changes in methylation, in particular reduced methylation in genes involved in immune function, metabolism, and on the X chromosome [75].

## 6. From Epigenetic Alterations to Chromosome Segregation Errors in Oocytes Exposed to BPA

An impact of BPA on the oocyte epigenome was confirmed by* in vitro* experiments using the same preantral follicle culture model in which nonlinear negative effects had been shown on spindle integrity, chromosome congression, and meiotic progression [38]. Follicles were chronically exposed* in vitro* to 3 or 300 nM BPA for 12 days, during which they matured under the influence of follicle stimulating hormone up to the large antral stage, when stimulation of ovulation by recombinant hCG and recombinant EGF caused resumption of oocyte maturation and development of oocytes to metaphase II, at day 13 of culture [38, 76]. Follicle survival and development, oocyte growth and maturation rates, and chromosome alignment on the metaphase II plate were compared between controls and BPA-exposed groups. Concomitantly, possible BPA-induced epigenetic alterations at the level of DNA methylation and posttranslational histone modifications were analysed in single oocytes. The specific culture conditions were shown not to affect the physiological DNA methylation pattern of maternally imprinted genes in the oocytes [77]. Thus, the methylation patterns of differentially methylated regions in the maternally imprinted,* Snrpn*,* Igf2r*,* Mest* genes and in the paternally imprinted* H19* gene were analysed using limiting dilution bisulfite pyrosequencing [78]. A cut-off of at least 50% abnormally methylated CpG sites was established to define epimutations.

Changes of posttranslational histone modifications had been previously imputed to BPA [79], and alterations of H3K9 trimethylation in pericentromeric heterochromatin had been associated in cultured oocytes to meiotic arrest, unaligned chromosomes, and spindle defects [80], a phenotype similar to that observed after treatment with a low BPA concentration [38]. Furthermore, biallelically different histone posttranslational epigenetic marks are functionally relevant for a correct expression of imprinted genes, as supported by the evidence that Beckwith-Wiedemann syndrome patients exhibit biallelic instead of monoallelic gene expression and similar marks for trimethylated histone H3 lysine 9 (H3K9me3) [81]. Based on these notions, relative histone H3K9 trimethylation and H4K12 acetylation were assessed by quantitative confocal microscopy of control or BPA-exposed mouse metaphase II oocytes from preantral follicle cultures.

Only the low BPA concentration (3 nM) caused a slight but significant acceleration of follicular growth. Overall, 7.5% of all analysed maternally imprinted alleles were abnormally demethylated in the group exposed to 3 nM BPA, a percentage significantly higher (*P* < 0.05) compared to the control and 300 nM BPA group [76]. The specific rates of abnormally demethylated alleles were 16.7% in* Mest*, 7.4% in* Igf2r*, and 4.8% in* Snrpn* alleles. No BPA effect was detected on the paternally imprinted* H19* allele. Single changes in cytosine methylation, presumably not relevant for gene expression, were not significantly affected by BPA exposures. The observations suggest that low, chronic BPA exposure during oocyte growth can either adversely influence maternal imprinting* per se* or affect imprint stability. The particular sensitivity of the maternal* Mest* allele to epimutations by low BPA concentrations may relate to influences of BPA on bidirectional signalling between the oocyte and its surrounding cumulus granulosa cells by gap junctional communication, as altered methylation of* Mest* was also detected in mouse oocytes of connexin 37 deficient transgenic mice [82].

**Figure 3:**
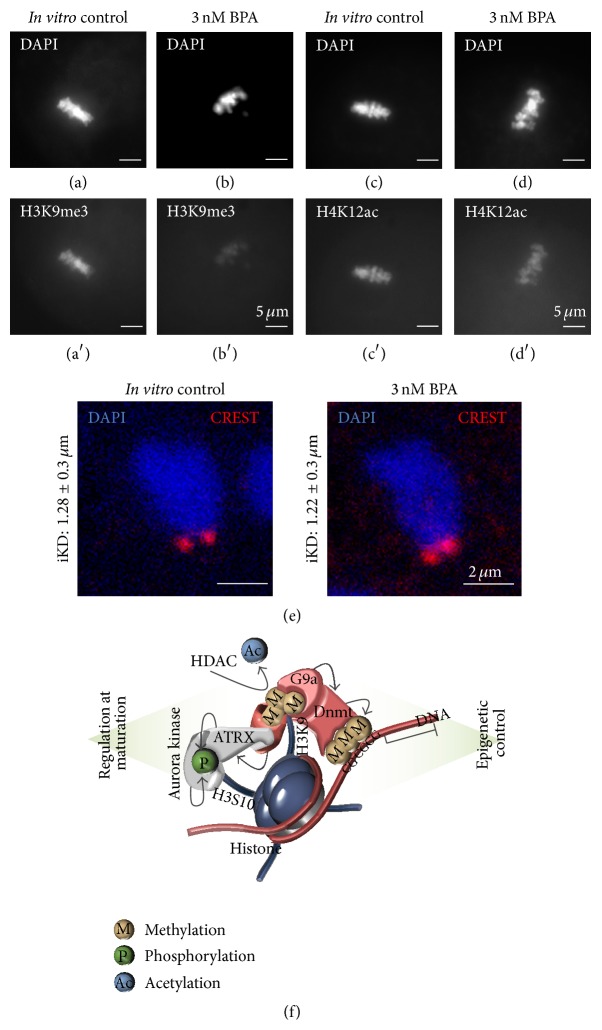
Changes in histone posttranslational modifications in low dose BPA-exposed metaphase II mouse oocytes. (a-b′) Images of histone H3K9 trimethylation in control and BPA-exposed oocytes: some unaligned chromosomes (b) and reduced trimethylated pericentromeric heterochromatin (b′) in the BPA group. (c-d′) Unchanged pattern of histone H4K12 acetylation. (e) Decreased distance between centromeres of sister chromatids in metaphase II chromosomes of BPA-exposed oocytes (blue) shown in fixed oocytes that were stained by CREST autoantibodies for centromeres (red). (f) Model indicating relevance of H3K9 trimethylation for recruitment of Dnmts (right side) and other factors like ATRX (ATP-dependent helicase that belongs to SWI/SNF family of chromatin remodelling factors) and Aurora kinase that might play a role in centromere regulation, microtubule attachment, and chromosome alignment through phosphorylation of different target proteins and histone H3S10 (left side). For further explanation, see text and [76].

After exposure to 3 nM BPA, congression failures and loosely aligned chromosomes at the metaphase II plate were observed (Figures 3(a) and 3(b)). Concomitantly, the relative H3K9me3 fluorescence was significantly lower compared to the control group (*P* < 0.001) (Figures 3(a′) and 3(b′)), whereas there was no difference in the intensity of fluorescence associated with H4K12 acetylation (Figures 3(c′) and 3(d′)). Interestingly, under the same treatment conditions, the average interkinetochore distance was slightly, but significantly, reduced from 1.28 ± 0.3 *μ*m to 1.22 ± 0.3 *μ*m (Figure 3(e)). H3K9 is acetylated in immature Germinal Vesicle pig oocytes but becomes deacetylated and trimethylated during meiosis I and at metaphase II [83]. Trimethylated H3K9 is also a hallmark of heterochromatin of mature metaphase II oocytes in the mouse and only gradually disappears from chromatin after fertilization, to increase again later in preimplantation development [57]. One can speculate that the changes in histone methylation of metaphase II chromosomes, induced by low chronic BPA treatment, may influence recruitment of DNA methyltransferases and chromatin remodelling proteins, like ATRX or Aurora kinase, that are critical for heterochromatin formation at centromeres (schematically depicted in Figure 3(f)), thereby affecting microtubule attachment and chromosome segregation [76]. In other words, the changes in histone posttranslational modification and DNA methylation detected in oocytes after BPA exposure could represent the “missing link” explaining the effects of low BPA concentrations on chromosome meiotic segregation.

## 7. Conclusions

By now there is increasing evidence that low BPA concentrations adversely affect the epigenome of mammalian female germ cells, with functional consequences on gene expression, and oocyte development and quality. There are specific time windows, during which profound chromatin remodelling occurs and maternal imprints are established or protected that appear particularly vulnerable to epigenetic deregulation by BPA: these correspond to primordial germ cell formation and oocyte meiotic prophase in the foetus, oocyte maturation after puberty, and early preimplantation development after fertilization. Transgenerational effects have also been observed in the offspring of BPA-treated rodents, although the epigenetic mechanisms of inheritance still need to be clarified. Thus BPA exposure might have long-lasting consequences on the female reproductive health, ranging from reduced fertility to offspring defects. Finally, the studies on histone posttranslational modifications suggest that BPA can also predispose the oocytes to altered chromosome behaviour in meiosis, particularly at low concentrations. The relevance of these findings for human health protection still needs to be fully assessed, but they warrant further investigation in both experimental models and humans.
